# A Meta-Analysis on the In Vitro Antagonistic Effects of Lactic Acid Bacteria from Dairy Products on Foodborne Pathogens

**DOI:** 10.3390/foods14060907

**Published:** 2025-03-07

**Authors:** Yara Loforte, Nathália Fernandes, André Martinho de Almeida, Vasco Cadavez, Ursula Gonzales-Barron

**Affiliations:** 1 CIMO, LA SusTEC, Instituto Politécnico de Bragança, Campus de Santa Apolónia, 5300-253 Bragança, Portugal; yara_loforte@hotmail.com (Y.L.); nathalia@ipb.pt (N.F.); vcadavez@ipb.pt (V.C.); 2 Divisão de Agricultura, Instituto Superior Politécnico de Manica, Campus de Matsinho, Manica 417, Mozambique; 3 LEAF-Linking Landscape, Environment, Agriculture and Food Research Center, Instituto Superior de Agronomia, Universidade de Lisboa, Tapada da Ajuda, 1349-017 Lisbon, Portugal; aalmeida@isa.ulisboa.pt; 4 Associate Laboratory TERRA, Instituto Superior de Agronomia, Universidade de Lisboa, Tapada da Ajuda, 1349-017 Lisbon, Portugal

**Keywords:** biopreservatives, dairy products, inhibition diameter, antimicrobial activity, random-effects model, meta-regression

## Abstract

Raw milk and traditional fermented foods such as artisanal cheese represent a natural source of lactic acid bacteria (LAB). They can produce antimicrobial compounds, such as bacteriocins and lactic acid, which may be exploited in dairy biopreservation. This study aimed to conduct a systematic review and meta-analysis to synthesize the inhibition diameter (ID) of LAB against *L. monocytogenes*, *S. aureus*, and *Salmonella* spp. Literature electronic searches were performed on PubMed, Scopus, and Web of Science, to identify articles that reported data on in-vitro antimicrobial activity by LAB isolated from dairy foods. A total of 1665 papers were retrieved, and 20 primary studies were selected according to the selection criteria, of which 397 observations were extracted. Random-effects meta-regression models were employed to describe the effects of LAB genus, pathogen concentration, susceptibility method, incubation time, inoculation volume, agar type and pH on the IDs for *L. monocytogens*, *S. aureus*, and *Salmonella* spp. *L. monocytogens* was the most susceptible pathogen (*p* < 0.05) to the LAB effects, followed by *S. aureus* and *Salmonella* spp. As a whole, LAB from the *Lacticaseibacillus* genus were the most effective (*p* < 0.05) in inhibiting *L. monocytogens* (21.49 ± 2.654 mm), followed by *S. aureus* (21.06 ± 2.056 mm). *Salmonella* spp. presented higher (*p* < 0.05) susceptibility to *Lactobacillus* genus (19.93 ± 2.456 mm). From the results, a general trend could be observed for the well-diffusion method to produce higher *(p* < 0.05) ID estimates than the spot and disk methods (30.73 ± 2.530 mm vs. 21.98 ± 1.309 mm vs. 13.39 ± 1.403 mm for *L. monocytogenes*; 22.37 ± 1.073 mm vs. 14.91 ± 2.312 mm vs. 20.30 ± 2.319 mm for *Salmonella* spp.), respectively. Among the tested moderators, the pathogen’s inoculum concentration, the in vitro susceptibility assay itself, incubation time and inoculation volume on agar are determinant parameters to be looked at when designing a robust and reproducible experimental plan. The in vitro results reinforced that LAB can be useful in controlling the development of pathogenic bacteria frequently found in the dairy industry.

## 1. Introduction

Milk and dairy products are among the most important foods consumed worldwide, providing essential nutrients [1]. However, these products can also be significant sources of harmful microorganisms. Common foodborne pathogens associated with them include *Listeria monocytogenes*, *Staphylococcus aureus*, and *Salmonella* spp. [2,3,4,5,6]. Farmed dairy animals serve as a major reservoir for these pathogens [6,7], and raw milk, in particular, provides an ideal growth medium for spoilage and pathogenic microorganisms, especially under inadequate manufacturing practices (e.g., poor hygienic practices or cross-contaminations) [6,8,9]. Factors such as inadequate refrigeration temperature, high pH and high water activity (Aw) during storage create an ideal environment for the growth of harmful bacteria [10,11,12]. The ability of *L. monocytogenes* to grow and multiply at refrigerated temperatures poses a significant challenge to both public health and the dairy industry [13,14,15,16]. Ensuring the safety and quality of milk and dairy products is therefore crucial, as contamination can occur at any stage of production, distribution, storage, preparation, or consumption [17].

Foodborne illnesses are a major global public health concern, responsible for approximately 600 million cases and 420,000 deaths annually due to the consumption of unsafe food [18,19,20,21]. In 2022, the most frequently reported foodborne pathogens in the European Union (EU) were *Campylobacter*, *Salmonella*, *Yersinia*, Shiga toxin-producing *Escherichia coli* (STEC) and *L. monocytogenes*. Listeriosis is the second leading cause of foodborne disease-related deaths in Europe, with a high mortality rate of 20–30% worldwide, following salmonellosis [13,15,22]. Salmonellosis remains the most common cause of gastroenteritis, with an estimated incidence of 2.8 billion cases worldwide annually [7,23]. *Staphylococcus aureus* is another significant foodborne pathogen that produces staphylococcal enterotoxins (SE) and is associated with food poisoning outbreaks worldwide [5].

Reports from seven countries—The United States, Finland, Netherlands, England and Wales, Germany, Poland, and France—indicate that 1–5% of total bacterial outbreaks are linked to milk and dairy products [10]. Even pasteurized milk can provide a favorable environment for the growth of *L. monocytogenes* and *S. aureus*, particularly in soft cheeses [5,16]. In 2022, the overall occurrence of *L. monocytogenes* in ready-to-eat (RTE) milk products in the EU was 0.37%. This overall occurrence was 0.18% in pasteurized milk cheese, 1.3% in raw milk or low heat-treated milk cheese, and 0.87% in milk. Raw or low heat-treated sheep milk cheeses showed the highest occurrence of this food category, with 2.0% in soft and semi-soft cheese, and 2.4% in hard cheese [23]. A meta-analysis also reported significant prevalence rates for *L. monocytogenes* (12.84%) in goat’s milk cheeses, *S. aureus* (16.04%), and *Salmonella* spp. (5.91%) [24].

An effective approach available to the dairy industry to inhibit the growth of undesirable microorganisms and add value to their products is the use of lactic acid bacteria (LAB) and/or their antimicrobial metabolites as natural preservatives [25]. This method, known as biopreservation, uses natural microflora and/or their antibacterial products to extend shelf life and enhance the safety of foods [26,27]. The antimicrobial compounds produced by LAB include bacteriocins, organic acids, hydrogen peroxide, carbon dioxide and other antimicrobial substances such as low molecular weight metabolites (reuterin, reutericyclin, diacetyl, fatty acids), which inhibit the growth of spoilage and foodborne pathogens [19,28,29,30,31,32,33]. This approach effectively enhances both the quality and safety of food products while preserving their nutritional value.

LAB are Gram-positive, catalase-negative microorganisms and lactic acid producers commonly found in raw milk and traditional fermented foods [34,35]. Their antimicrobial activity is largely attributed to bacteriocins—ribosomally synthesized proteinaceous inhibitors known for their bactericidal and bacteriolytic effects against Gram-positive bacteria, including foodborne pathogens such as *L. monocytogenes* and *S. aureus*, as well as spoilage organisms [36,37,38,39,40,41,42]. The primary organic acids produced by LAB, such as lactic and acetic acid, lower the pH of the food environment, creating acidic conditions that are unfavorable for the growth of many pathogenic and spoilage bacteria. This is the main mechanism of biopreservation in fermented foods, contributing to enhanced stability and extended shelf life [19,30,43,44,45].

LAB are recognized by the U.S. Food and Drug Administration (FDA) as Generally Regarded as Safe (GRAS) [32,46,47]. Their application as natural food preservatives can reduce or eliminate the need for chemical preservatives, thereby avoiding the harmful side effects associated with these substances [19,48], which constitutes a challenge to modern food-processing technology [49]. Nisin (E234), produced by *Lactococcus lactis subsp. lactis*, was the first bacteriocin approved as a food preservative and remains the most extensively studied and commercially available [47]. It is widely used in the dairy industry, particularly in cheese production, due to its broad-spectrum activity against a wide range of Gram-positive bacteria, including *L. monocytogenes* and *S. aureus* [30,50,51,52,53].

In traditional raw milk cheese, LAB play a crucial role, either as starter cultures that rapidly acidify milk or as secondary microbiota that contribute significantly during the ripening process [35,44]. They promote desirable organoleptic properties, such as flavor and aroma development, through enzymatic, proteolytic, and lipolytic activities [19,28,33,36,54,55]. Additionally, they may produce exopolysaccharides (EPS) which improve texture and enhance safety through antimicrobial compounds [55,56].

Meta-analysis is a set of statistical methods used to synthesize, integrate, and compare findings from multiple primary studies investigating the same research question [57,58]. In meta-analyses, meta-regression analysis is often employed to evaluate the relative importance of one or more study characteristics (moderators) in the measured outcome or effect size [59]

Thus, the objective of this study was twofold: (1) to conduct a systematic review and meta-analysis to synthesize the in vitro antimicrobial activity of indigenous LAB isolated from dairy products against *L. monocytogenes*, *S. aureus*, and *Salmonella* spp.; and (2) to identify the study characteristics or moderators acting as sources of heterogeneity in LAB antimicrobial activity through a meta-regression analysis.

## 2. Materials and Methods

A systematic review is a robust method for summarizing primary research on a specific topic in a standardized and reliable way [60]. It involves comprehensive searches across multiple bibliographic databases to identify eligible studies [61]. The Preferred Reporting Items for Systematic reviews and Meta-Analyses (PRISMA) guidelines were used to perform this systematic review [62]. The research question guiding the data search was “Evaluate the effectiveness of LAB genus/species in their in vitro antimicrobial activity against three foodborne pathogens: *L. monocytogenes*, *S. aureus*, and *Salmonella* spp., and identify the most effective susceptibility testing method(s) and the potential moderators affecting the outcomes”.

### 2.1. Literature Review and Data Extraction

In November 2022, two authors conducted electronic searches in PubMed, Scopus, and Web of Science databases to identify original publications reporting data on the in vitro antimicrobial activity of LAB isolated from dairy foods against foodborne pathogens: *L. monocytogenes*, *S. aureus*, and *Salmonella* spp. The search was conducted in the title, abstract and keywords to identify original publications. The bibliographic searches were conducted by combining terms related to dairy foods, pathogens, and biopreservation using logical connectors “and” and “or” according to each database’s syntax. The following terms were used: (((cheese OR milk OR dairy) AND (lactic acid bacteria)) OR ((starter culture) OR (biocontrol) OR (*lactobacillus*))) AND ((antimicrobial*) OR (functional) OR (preserv*) OR (biopreserv*) OR (antagoni*)) AND ((*Salmonella*) OR (*Listeria monocytogenes*) OR (*Staphylococcus aureus*) OR (pathogen*)).

All bulk of references were added to a project built on the Rayyan software, no public version [63], and duplicates were identified and removed. The abstract of each reference was evaluated against the following pre-defined inclusion criteria: (1) the article must be a primary study written in English or Portuguese; (2) article published between 2000 and 2022; (3) the article must provide data on in vitro antimicrobial activity of LAB against at least one of the selected pathogens*;* (4) the article must indicate the LAB origin as dairy; and (5) both LAB and pathogen must be identified at the species level. At the full-text assessment level, the pre-selected articles had to provide the method employed for determining the inhibition diameter, and the sample size or number of repetitions (N).

Data and metadata were collected from the included studies. The information included: (1) a code for the primary study, (2) country of conduction of the experiment, (3) year, (4) the origin of the LAB strain (milk source [cow, goat, sheep, NA]; (5) food category [milk, cheese, yogurt]), (6) LAB and pathogens identification (species, genus and strain), (7) antimicrobial susceptibility method (agar methods [disk diffusion, well diffusion, or spot test]), (8) the mean inhibition diameter (mm) and the sample size or number of replicates, (9) temperature (°C) and time of incubation (h) of the plates, (10) agar medium [Brain Heart Infusion, Muller–Hilton, De Man–Rogosa–Sharpe, Nutrient and Trypticase Soy], (11) LAB concentration in the aliquot (log CFU/mL), (12) pathogen concentration in the agar (log CFU/mL), (13) aliquot volume of LAB suspension (μL), and (14) pH of LAB suspension.

### 2.2. Meta-Regression Modeling

For this meta-analysis, the *population* was defined as LAB isolated from dairy foods, while the *measured outcome* was the mean inhibition diameter (ID) in millimeters. The inhibition diameter included the zone occupied by the disk or the well. Three types of meta-regression models were fitted to the data according to the level of partitioning and objectives: (1) pooling meta-regression models to estimate pooled IDs by pathogen, by LAB and by susceptibility method; and (2) pathogen-specific meta-regression models to assess the relative importance of study characteristics and to conduct heterogeneity analysis by pathogen; and (3) an overarching meta-regression model with the same objective as in (2) yet using the full data.

#### 2.2.1. Pooling Meta-Regression Models

The meta-regression general model,

*ID_pgaj_* = a*Pathogen_p_* + b*LAB genus_g_* + c*Assay_a_* + d[*Pathogen*] + e*Time* + (f + *u_j_*) + *ε_pgaj_* (1)

was fitted to the whole data, where a, b, c are the fixed effect vectors of the p types of pathogens, the g types of LAB genus, and the a types of susceptibility assay, respectively; d is the effect of one-log increase in pathogen concentration (log CFU/mL), e the effect of incubation time (h), f the model’s intercept that is shifted by *u_j_*, the random effects due to the primary study *j* that contains the remaining between-study variability. The term *ε_pgaj_* represents the residuals and accounts for the sampling variability.

When fitted to the full data, the model allowed the comparison of the pooled inhibition diameters among the three pathogens. Afterward, the term a*Pathogen* was removed from Equation (1), and the resulting model was fitted to data subsets partitioned by pathogen. Each of the three models was employed to obtain pooled IDs by LAB genus and susceptibility assay. All comparisons were carried out on pooled IDs solved for a pathogen concentration of 7.0 log CFU/mL and an incubation time of 24 h in order.

#### 2.2.2. Pathogen-Specific Meta-Regression Models

The objective of the pathogen-specific meta-regressions was to evaluate which moderators or study characteristics drive the changes in the measured ID by a specific pathogen. Data were first partitioned by pathogen, and the following meta-regression models,

*ID_gaj_* = a*LAB genus_g_* + b*Assay_a_* + c[*Pathogen*] + d*Time* + d*Vol* + (f + *u_j_*) + *ε_gaj_* (2)

*ID_gaj_* = a*LAB genus_g_* + b*Vol* (*Assay*)*_a_* + c[*Pathogen*] + d*Time* + (f + *u_j_*) + *ε_gaj_* (3)

*ID_gamj_* = a*LAB genus_g_* + b*Assay_a_* + c*Agar_m_* + (f + *u_j_*) + *ε_gamj_* (4)

were adjusted to the datasets of *L. monocytogenes*, *S. aureus* and *Salmonella* spp., respectively. The coefficients can be interpreted as in Equation (1); and, in addition, in Equation (3), b is the set of slopes for the inoculum volume (*Vol*) nested within susceptibility assay (*Assay*), and in Equation (4), b is the set of fixed effect for the types of agar (*Agar*). Note that the three models have different moderators since they are driven by datasets of different structures, and the non-significant terms were dropped at α = 0.10.

#### 2.2.3. Overarching Meta-Regression Model

The final or overarching meta-regression model used the full dataset to identify the moderators that were more determinant in explaining the between-study variability in ID. The dataset was described using a meta-regression model of the form,

*ID_pgaj_* = a*Pathogen_p_* + b*LAB genus_g_* + c*Assay_a_* + d[*Pathogen*] + e*Time* + f*Vol* (*Assay*)*_a_* (f + *u_j_*) + *ε_pgaj_* (5)

The model’s coefficients, random effects and error terms are defined as in previous equations.

#### 2.2.4. Heterogeneity and Publication Bias

Heterogeneity refers to the variability in effect sizes across studies included in a meta-analysis. The magnitude of heterogeneity across the studies was quantified by the intraclass correlation *I*^2^, which is the proportion of total variability attributable to between-study differences. First, from the intercept-only model, the values of within-study variability (*s*^2^) and between-study variability (*τ*^2^) were obtained. The intra-class correlation (*I*^2^) was then calculated as,

(6) $$I^{2}=\frac{\tau^{2}}{\left(\tau^{2}+s^{2}\right)}\times 100\%$$

The *I*^2^ scale ranges from 0 to 100% [59,64,65,66]. An *I*^2^ value close to 0% indicates no heterogeneity between studies (homogeneity). *I*^2^ values of up to 25%, 50% and 75% are considered low, moderate, and high heterogeneity, respectively [66].

As a next step, the model with moderators is fitted, and the residual between-study variability ($\tau_{res}^{2}$) is obtained. Next, the between-study variability explained by significant moderators (*R*^2^) is determined as,

(7) $$R^{2}=\frac{\left(\tau^{2}-\tau_{res}^{2}\right)}{\tau^{2}}$$

Publication bias refers to the tendency for studies with smaller effect sizes or negative results to be less likely published or included in the analysis [65], thereby influencing the pooled results obtained from the meta-analysis. Publication bias was assessed for every meta-regression by two methods: (1) Building funnel plots for visually assessing the degree of asymmetrical distribution of data points, signaling the likely presence of publication bias [59,64,65]; and (2) by incorporating the sample size N as a moderator, and assessing whether the *p*-value is lower than 0.05, signaling the likely presence of publication bias.

All meta-analysis regressions, estimations and comparisons between pooled IDs were carried out in the R software version 4.3.3 [67] using the metafor package version: 4.8-0 [68,69,70].

## 3. Results and Discussion

### 3.1. Description of the Meta-Analytical Data

**Figure 1:**
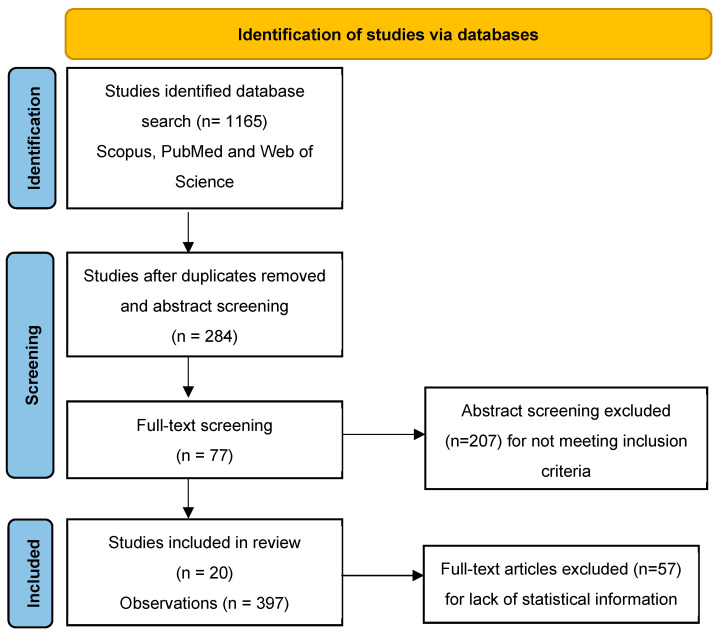
PRISMA flow diagram of study selection.

A total of 1165 articles were initially retrieved from the databases. After deduplication, the titles and abstracts of the remaining articles were screened, leading to 284 articles selected for full-text review. Of these, 77 full-text articles were retrieved, and finally, 20 primary studies were considered eligible for inclusion in the meta-analysis, since they met all criteria and provided therefore sufficient extractable data. This process resulted in 397 observations available for analysis. A meta-analysis of different pathogen groups included 15 primary studies, consisting of 165 observations [71,72,73,74,75,76,77,78,79,80,81,82,83,84,85] for *L. monocytogenes*, 17 primary studies, with 171 observations [71,72,73,74,76,77,79,80,81,82,83,84,86,87,88,89,90] for *S. aureus*; and 8 primary studies, with 61 observations [71,73,74,77,80,82,84,89] for *Salmonella* spp. The study selection process, as per PRISMA guidelines [91], is summarized in Figure 1.

### 3.2. Pooled Inhibition Diameters

The IDs produced by LAB were pooled using meta-regressions, and the resulting estimates are presented in Table 1. At an inner level of analysis, pooled IDs are provided by susceptibility assay (i.e., disk-diffusion, well-diffusion, or spot test). In all cases, the pooled IDs shown in Table 1 were estimated at a pathogen concentration of 7.0 log CFU/mL and an incubation period of 24 h, in order to allow comparisons.

At the most outer level of analysis, it was found that *L. monocytogenes* was the most susceptible bacterium (*p* < 0.05) to the antimicrobial effects of LAB, followed by *S. aureus* and *Salmonella* spp. (Table 1). The presence of an outer membrane (OM) is one of the features that differentiate Gram-negative from Gram-positive bacteria [92].

This OM, composed of a double layer of phospholipids and lipopolysaccharides (LPS), acts as an efficient permeability barrier. It reduces permeability and regulates the passage of metabolites, including antimicrobial compounds such as bacteriocins, into the cell [93,94,95,96,97]. The reduced antimicrobial effect of LAB against Salmonella spp., may be linked to the presence of this OM, as it limits the penetration of antimicrobial agents.

The predominant genera of LAB identified in this study were *Lactobacillus* (n = 128), followed by *Lacticaseibacillus* (n = 97), *Lactococcus* (n = 89), *Enterococcus* (n = 75), and *Leuconostoc* (n = 8). This finding is corroborated by previous studies that have described the most common LAB genera in milk microbiota, including *Bifidobacterium*, *Enterococcus*, *Lacticaseibacillus*, *Lactobacillus*, *Lactococcus*, *Leuconostoc*, *Pediococcus*, *Carnobacterium*, *Aerococcus*, *Tetragenococcus*, *Vagococcus*, *Oenococcus*, *Sporolactobacillus*, *Weissella and Streptococcus* [30,52,98].

The pooled IDs produced by LAB (Table 1), categorized by pathogen, revealed that *Lacticaseibacillus* (21.49 ± 2.654 mm), *Lactobacillus* (20.36 ± 2.649 mm) and *Enterococcus (*20.13 ± 2.213 mm) were the most effective in inhibiting *L. monocytogenes.* Although *Lacticaseibacillus* exhibited numerically higher IDs, the differences among these three genera were not statistically significant (*p* > 0.05). Meanwhile, LAB from other genera, such as *Leuconostoc* (17.51 ± 2.678 mm) and *Lactococcus* (16.17 ± 2.660 mm), showed the lowest inhibition values. While no significant differences were found within the most or least effective LAB groups (as indicated by the equal superscript uppercase letters), significant differences were observed when comparing these two groups to each other.

Regarding *S. aureus*, *Lacticaseibacillus* (21.06 ± 2.056 mm), *Lactobacillus* spp. (20.02 ± 2.054 mm) and *Enterococcus* spp. (19.85 ± 2.065 mm) were again the most effective in inhibiting this pathogen. Conversely, *Leuconostoc* and *Lactococcus* exhibited the lowest antimicrobial activity, with pooled IDs of 17.97 ± 2.079 mm and 15.98 ± 2.089 mm, respectively. This trend is consistent with the inhibition observed for *L. monocytogenes* meta-regression, suggesting that LAB exhibited a similar mode of action against both Gram-positive bacteria.

*Salmonella* spp. showed significantly higher susceptibility to the *Lactobacillus* (19.93 ± 2.456 mm), and *Lacticaseibacillus* (19.06 ± 2.467 mm), with no significant differences observed between their IDs. However, *Lactococcus* displayed the lowest pooled ID (11.98 ± 2.565) against this pathogen.

The meta-analysis also exposed that the susceptibility assay may have an effect on the inhibitory activity measured. The results indicate that the well diffusion assay tended to generate higher IDs (Table 1) compared to the spot and agar disk-diffusion methods. For instance, the pooled IDs of *Lactobacillus* against *L. monocytogenes* were 30.73 ± 2.530 mm, 21.98 ± 1.309 mm, and 13.39 ± 1.403 mm for well-diffusion, spot test and disk-diffusion methods, respectively. Similarly, for *Salmonella* spp., the values were 22.37 ± 1.073 mm, 13.29 ± 2.317 mm, and 20.30 ± 2.319 mm, respectively. Only in the model adjusted for *S. aureus* did the agar disk-diffusion assay result in a higher pooled ID of 27.28 ± 2.730 mm, compared to the well-diffusion (23.40 ± 2.107 mm) and spot test (17.26 ± 2.793 mm) methods, specifically for *Lacticaseibacillus*.

In the agar well diffusion method, a defined volume of the antimicrobial agent is placed directly into a well, typically measuring 6–8 mm in diameter, on the agar plate. This approach maximizes the contact area between the antimicrobial agent and the agar surface, resulting in a larger zone of inhibition compared to methods where the agent is applied directly as disk-diffusion or spot test [99]. Similarly, in the agar disk-diffusion method, a predefined amount of antimicrobial agent contained in a filter paper disk is placed on the surface of an agar plate previously inoculated with a standardized inoculum of the indicator microorganism [100]. Both methods are unable to distinguish between bactericidal and bacteriostatic effects [99]. On the other hand, in the spot test, a small volume of the antimicrobial agent is applied directly to the surface of an agar plate to evaluate antimicrobial activity [97]. In all methods, plates are incubated under appropriate conditions to allow the growth of the test microorganism. The principle behind these methods is that the antimicrobial substance diffuses through the agar to inhibit the growth of the indicator microorganism, which is typically observed as clear zones or “inhibition halos” around the colonies. These inhibition zones can be measured either as the diameter or area [42,99,101,102,103,104,105]. The size of the inhibition zone is influenced by both the diffusion rate of the antimicrobial substance and the growth rate of the indicator organism [106].

### 3.3. Meta-Regression Models

#### 3.3.1. Listeria Monocytogenes

The estimates of the meta-regression model on IDs produced by LAB against *L. monocytogenes* are presented in Table 2. The moderator LAB genus, susceptibility method, inoculation volume, and incubation time were kept in the model solution as they were all significant. According to this meta-regression, LAB belonging to the *Enterococcus* genus achieved the highest inhibition against *L. monocytogenes* (intercept estimate of 11.26 ± 1.687 mm). This finding is consistent with the literature on the effectiveness of enterocins—antimicrobial peptides produced by the *Enterococcus* genus—which are particularly notable for their broad-spectrum activity against closely related Gram-positive bacteria, including spoilage and pathogenic species such as *L. monocytogenes* [27,107,108,109,110]. Significant differences in IDs were observed across different LAB genera (*p* < 0.001). Compared to *Enterococcus*, the pooled IDs were lower by −3.408 ± 0.317 mm for *Lactobacillus*, by −5.120 ± 0.607 mm for *Lactococcus*, by −5.906 ± 0.701 mm for *Leuconostoc*, and by −6.192 ± 0.549 mm for *Lacticaseibacillus*.

The genus *Enterococcus* belongs to the most studied LAB [27] and constitutes the third main genus, after *Lactobacillus* and *Streptococcus* [111]. They are commonly found in dairy products, particularly in traditional cheeses, where they are used as starter cultures or protective cultures, due to their biotechnological traits, including enzymatic and proteolytic activities, as well as their production of antimicrobial compounds such as enterocins, lactic acid and hydrogen peroxide [28,107,112,113,114]. Their metabolites significantly contribute to the development of organoleptic properties during cheese ripening [111,113,115,116]. Several enterocins have been evaluated for their effectiveness in inhibiting *Listeria* spp. in dairy systems, where *Enterococcus* are often isolated as desirable microflora [109,117]. Lactic acid, the primary product of glucose fermentation [107], contributes to pH reduction, thereby limiting the growth of spoilage and pathogenic bacteria. It plays a crucial role in the preservation of artisanal cheese [45,101,118]. Hydrogen peroxide (H_2_O_2_) is produced as a metabolic byproduct during the fermentation processes of LAB, exerting antimicrobial activity through oxidative damage to proteins and increased membrane permeability [28]. Furthermore, *Enterococcus* exhibits resistance to the pasteurization process at lower temperatures, and can adapt to various substrates and growth conditions, including a wide range of temperatures, pH levels, and salinity [119,120].

**Figure 2:**
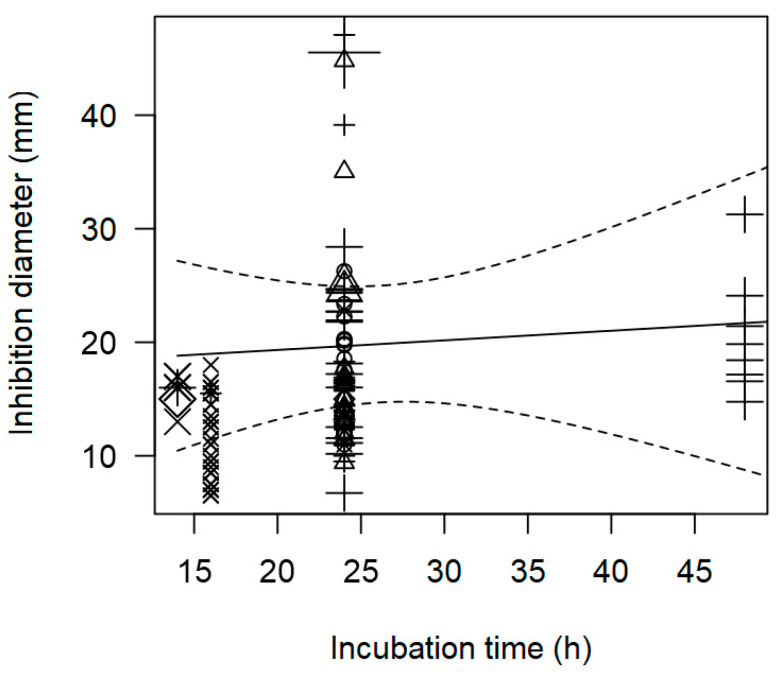
Scatter plot depicting the effect (*p* < 0.001) of incubation time on the inhibition diameters against *L. monocytogenes.* Markers symbolize LAB genus: ○ = *Enterococcus*, ∆ = *Lacticaseibacillus*, + = *Lactobacillus*, × = *Lactococcus*, ◊ = *Leuconostoc*; and marker size is proportional to study size.

Regarding the susceptibility test method, the well diffusion method was confirmed to result in greater ID values than the disk diffusion method (*p* < 0.001). The variables inoculation volume and incubation time (*p* < 0.001, Table 2) showed significant positive correlations with the IDs, as increased inoculum size and longer incubation times were specifically associated with increased efficacy of LAB in suppressing *L. monocytogenes* growth. As observed in the bubble plot (Figure 2), there is a trend where the IDs increase as the incubation time (h) increases (*p* < 0.001). Additionally, longer incubation periods and higher IDs were notably linked to the *Lactobacillus* genus. The susceptibility of antimicrobial agents is also influenced by the inoculation size, emphasizing the importance of standardized protocols in susceptibility testing of LAB [121]. Inoculum size should be defined for each developed method, as comparisons between results are often hindered due to variations in non-standardized factors such as inoculum preparation techniques, inoculum size, growth medium, and incubation conditions [99,122].

**Figure 3:**
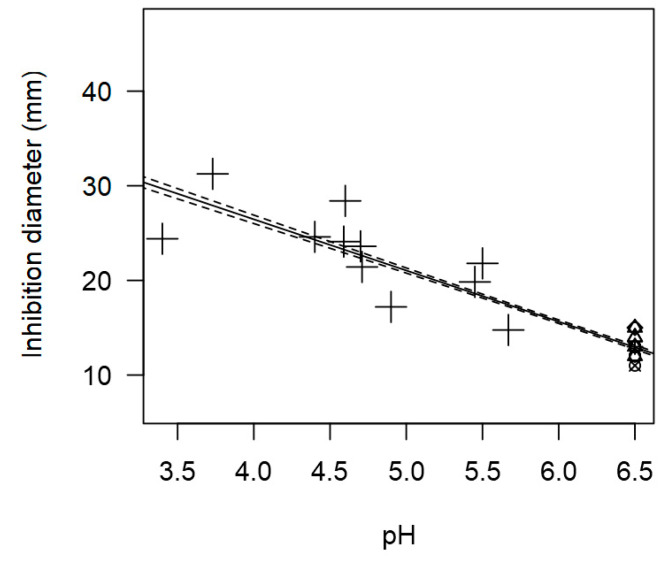
Scatter plot depicting the effect (*p* < 0.001) of pH on the inhibition diameters against L. monocytogenes. Marker size is proportional to study size; and symbolizes LAB genus: ○ = *Enterococcus*, ∆ = *Lacticaseibacillus*, + = *Lactobacillus*, × = *Lactococcus*. pH was not added in the final model as a moderator because it had too few incidences.

Due to the low availability of pH information in the *L. monocytogenes* dataset, pH was not included in the meta-regression model as a moderator. However, the bubble plot (Figure 3) shows a significant inverse correlation between pH and the measured IDs (*p* < 0.001).

Notably, as pH increases, IDs decrease, suggesting that higher pH levels in the suspension may reduce the effectiveness of the *Lactobacillus* genus. The preservative effect exerted by LAB is primarily due to the production of organic acids, such as lactic acid, which contributes to a lower pH [97]. Consequently, the inhibitory effect of LAB on *L. monocytogenes* is enhanced under more acid conditions (pH < 5.0). The finding is consistent with previous studies in blue cheese production, showing that *L. monocytogenes* increased by 0.58 to 1.22 log_10_ cfu/g during the first 24 h of the cheesemaking process. The pathogen’s growth was halted when the cheese’s pH dropped below 5.0. In this context, acidification at the beginning of cheesemaking, reaching a pH between 4.6 and 4.9 during the first 20 days, is crucial for reducing the pathogen’s population [123].

In the *L. monocytogenes* meta-regression model, heterogeneity analysis revealed that 99.9% of the variability between studies (τ^2^) could be explained by the moderators—LAB genus, susceptibility test method, inoculum volume and incubation time. This suggests that these variables can almost entirely account for the differences in IDs observed across the studies. This outcome only reinforces the importance of establishing a standardized protocol defining an optimal assay, inoculation volume and incubation time, since these three factors strongly affect the inhibition zone formed and measured.

**Figure 4:**
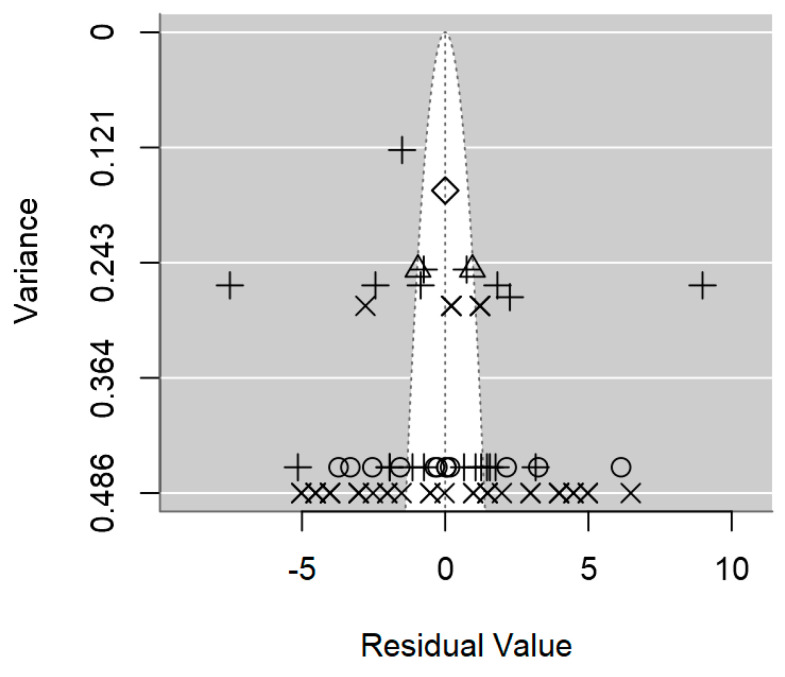
Funnel plot of the meta-regression model on the inhibition diameter produced by lactic acid bacteria against *L. monocytogenes*. Markers symbolise bacterium: ○ = *Enterococcus*, ∆ = *Lacticaseibacillus*, + = *Lactobacillus*, × = *Lactococcus*, ◊ = *Leuconostoc*.

A funnel plot is a valuable visual tool commonly used in meta-regression to evaluate the presence of publication bias. In the absence of publication bias, larger studies will cluster around the average, while smaller studies will be symmetrically distributed on both sides of the average. As illustrated in Figure 4, the symmetrical distribution of data in the plot hints at the absence of publication bias, which is also supported by the non-significant *p-*value of 0.509 (Table 2).

#### 3.3.2. *Staphylococcus aureus*

The meta-regression on LAB antimicrobial activity against *S. aureus* was fitted using LAB genus, pathogen concentration (log CFU/mL), incubation time (h), and inoculation volume (μL) with susceptibility test assay as a moderator (Table 3). Similarly to the meta-regression findings for *L. monocytogens*, it was observed that *Enterococcus* was again the most effective LAB genus (91.63 ± 2.518 mm, *p* < 0.001) in inhibiting this pathogen, which can be deduced from the higher intercept for ID; meanwhile, the genera *Leuconostoc*, *Lactococcus*, *Lactobacillus*, and *Lacticaseibacillus* did not present significant differences in their capability to inhibit *S. aureus*.

**Figure 5:**
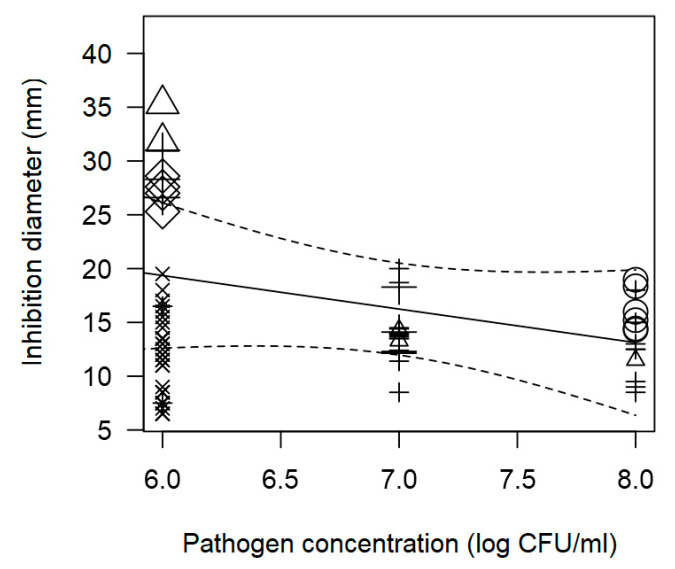
Scatter plot depicting the effect (*p* < 0.001) of pathogen concentration on the inhibition diameters against *S. aureus.* Markers symbolize LAB genus: ○ = *Enterococcus*, ∆ = *Lacticaseibacillus*, + = *Lactobacillus*, × = *Lactococcus*, ◊ = *Leuconostoc*; and marker size is proportional to study size.

As with the *L. monocytogenes* meta-regression, the *S. aureus* model also demonstrated that factors such as pathogen concentration, and incubation time significantly influence the antibacterial effectiveness of LAB against *S. aureus* (*p* < 0.001). There is a clear inverse association between ID produced by LAB and pathogen concentration: as the pathogen concentration increases, the effectiveness of LAB in inhibiting pathogen growth decreases. This trend is illustrated in the scatter plot (Figure 5), where higher pathogen concentrations (8 log CFU/mL) result in lower inhibition values (<15 mm).

**Figure 6:**
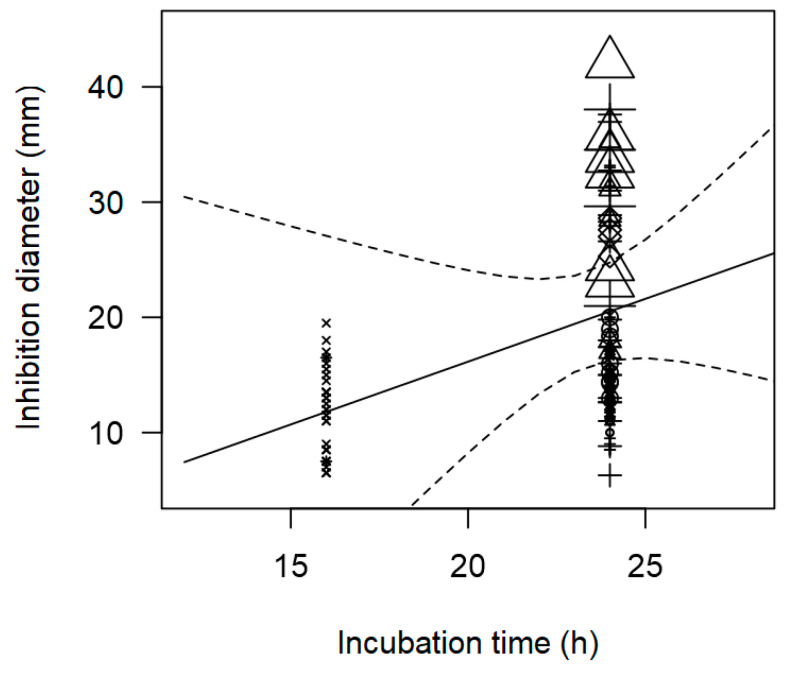
Scatter plot depicting the effect (*p* < 0.001) of incubation time on the inhibition diameters against *S. aureus*. Markers symbolize LAB genus: ○ = *Enterococcus*, ∆ = *Lacticaseibacillus*, + = *Lactobacillus*, × = *Lactococcus*, ◊ = L*euconostoc*; and marker size is proportional to study size.

The study found a positive and significant correlation between incubation time and inhibition values (*p* < 0.0001, Table 3). This indicates that an incubation period of at least 24 h leads to higher inhibition values (>20 mm, Figure 6). These results are consistent with the literature, which suggests that factors affecting the growth and production of bacteriocins include incubation time. A minimum of 24 h is required to allow the development of indicator zones using plating methods. Overnight incubation is generally the minimum time required before inhibition zones can be accurately measured [42].

Concerning the inoculation volume (μL), its effect on the extent of inhibition depended on the susceptibility test assay; thus, the agar spot method (10.65 ± 0.356, *p* < 0.0001) was linked to a greater effect of inoculation volume than the agar well-diffusion method (0.675 ± 0.019, *p* < 0.0001). However, in both cases, the greater the inoculation volume, the greater the inhibition zone formed on the agar.

**Figure 7:**
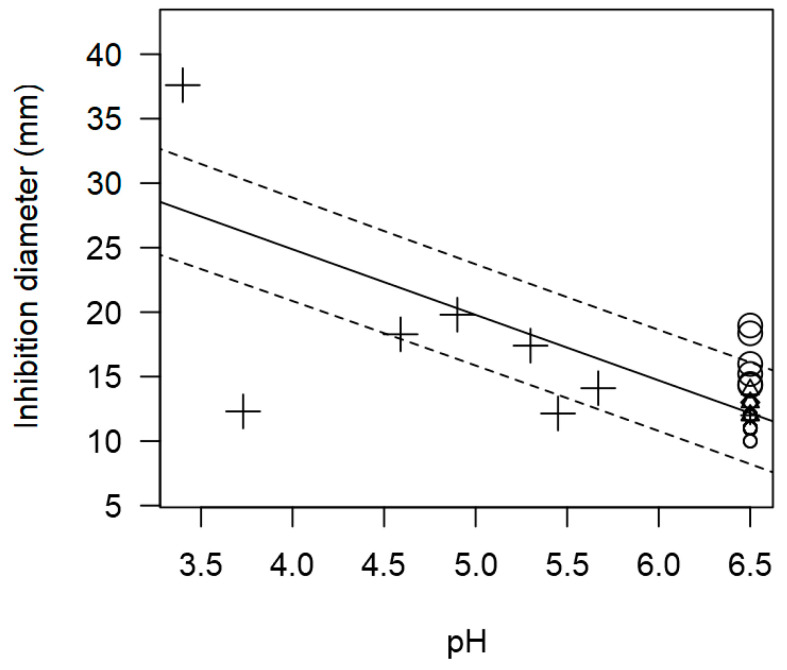
Scatter plot depicting the effect (*p* < 0.001) of pH on the inhibition diameters against *S. aureus*. Markers symbolize LAB genus: ○ = *Enterococcus*, ∆ = *Lacticaseibacillus*, + = *Lactobacillus*, × = *Lactococcus*, ◊ = *Leuconostoc;* and marker size is proportional to study size. pH was not added in the final model as a moderator because it had too few incidences.

Regarding pH, similar to the findings from the meta-regression for *L. monocytogenes*, this variable could not be included as a moderator in the meta-regression model due to its low incidence. However, the scatter plot (Figure 7) once again demonstrates a significant effect of pH (*p* < 0.001) on the IDs against *S. aureus*. The observed trends indicate that as the pH decreases, the ID generally increases. For instance, *Lactobacillus* genus exhibited higher inhibition values (>20 mm, Figure 7) under more acid conditions (*p* < 5.0).

The pH, which refers to the local concentration of hydrogen ions (H^+^), is one of the most significant environmental parameters impacting pathogen growth [124]. Organic acids such as lactic and acetic acid, which are primary byproducts of LAB metabolism, are essential for lowering the pH in fermented dairy products. This increase in acidity enhances the antimicrobial effectiveness of LAB. Lower pH levels create an environment that is unfavorable for many pathogenic and spoilage microorganisms, thereby improving food safety and shelf life [125]. These findings are consistent with those of other researchers who have reported that pH is the main environmental factor impairing the growth of *S. aureus* and the production of SE in cheese [5].

The heterogeneity analysis showed that the intraclass correlation was moderate (~50%) and, furthermore, that the incorporation of LAB genus, pathogen concentration, incubation time, and inoculation volume (μL) by susceptibility test assay could account for 99.9% of the variability between studies (Table 3).

**Figure 8:**
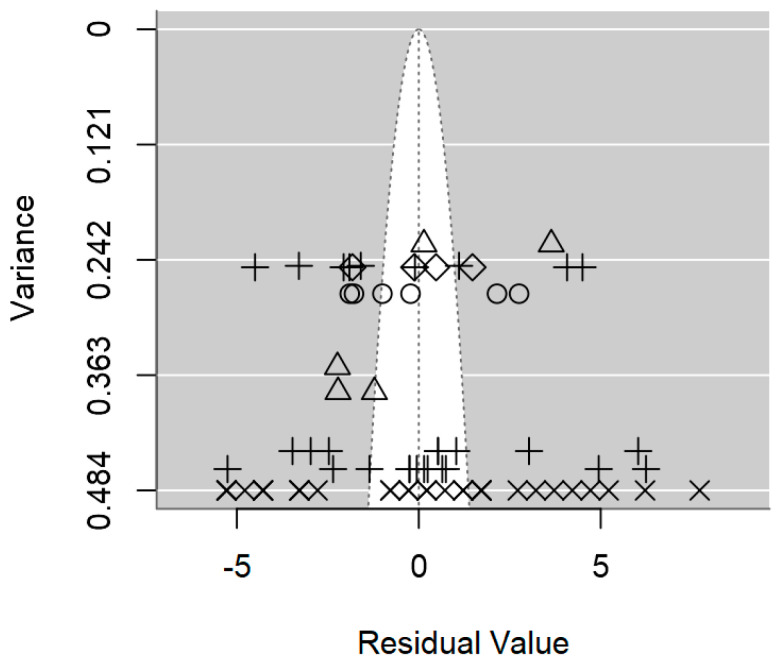
Funnel plot of the meta-regression model on the inhibition diameter produced by lactic acid bacteria against *S. aureus*. Markers symbolise bacterium: ○ = *Enterococcus*, ∆ = *Lacticaseibacillus*, + = *Lactobacillus*, × = *Lactococcus*, ◊ = *Leuconostoc*.

The funnel plot revealed a non-symmetrical distribution of studies around the mean effect size, with a notable absence of studies in the middle-right area, thereby signaling the likelihood of publication bias (Figure 8). Moreover, the formal test for assessing bias indicated the presence of publication bias (*p* = 0.001; Table 3). This suggests that smaller studies with non-significant findings may have been unpublished [69].

#### 3.3.3. *Salmonella* spp.

The meta-regression model for *Salmonella* spp. included the LAB genus, susceptibility test assay, and agar type. Note that the *Salmonella* data partition contained fewer LAB genera. The results on ID are presented in Table 4. Higher IDs were associated with the *Lacticaseibacillus* (17.88 ± 2.978 mm for the intercept, *p* < 0.0001), which did not differ significantly from the Lactobacillus in the antimicrobial activity against *Salmonella* spp. Among the three genera, Lactococcus possessed the lower action against this pathogen (*p* = 0.006). Halos measured on nutrient agar (11.91 ± 5.481 mm, *p* = 0.029) were linked to greater values in comparison to milk (*p* = 0.211) and MRS agar (*p* = 0.256), whereas lower IDs were linked to the disk diffusion assay (*p* = 0.032).

In this model, the intraclass correlation was moderate (I^2^ = 57.2%), and the inclusion of moderators explained 55.4% of the between-study variability. Unlike the other two regression models, this meta-regression left some residual variability unaccounted for, probably arising from the fewer observations recovered.

**Figure 9:**
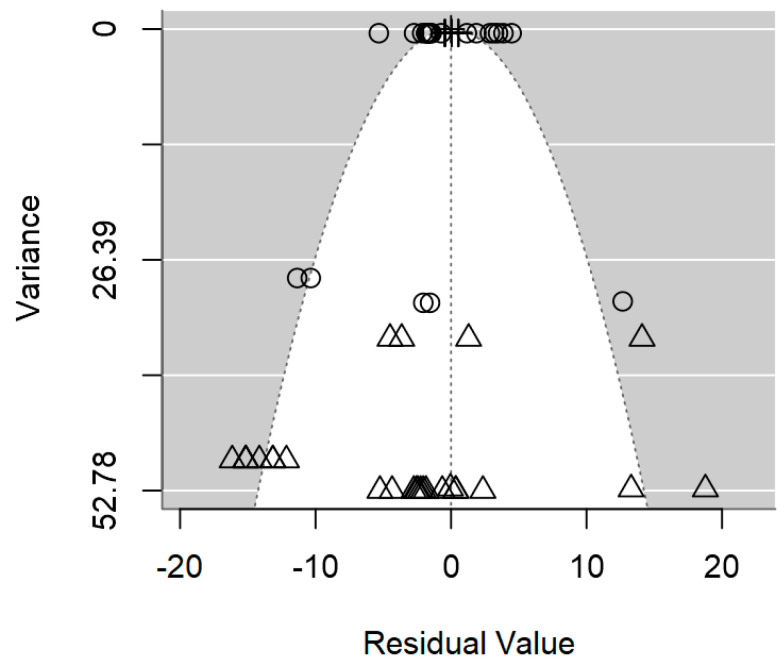
Funnel plot of the meta-regression model on the inhibition diameter produced by lactic acid bacteria against *Salmonella* spp. Markers symbolise bacterium: ○ = *Lacticaseibacillus*, ∆ = *Lactobacillus*, + = *Lactococcus*.

A funnel plot is a widely used visual tool in meta-regression analyses to assess the presence of publication bias. In the absence of publication bias, larger studies will cluster around the average, while smaller studies will be symmetrically distributed on both sides of the average [59]. However, in the case of *Salmonella* spp., the funnel plot in Figure 9 exhibits an asymmetrical distribution of outcomes, meaning that studies with smaller sample sizes are likely underrepresented in the published literature. This finding aligns with the significant evidence of publication bias demonstrated by the meta-regression (*p* < 0.0001; Table 4). 

### 3.4. Overarching Meta-Regression Model

The outcomes of the overarching meta-regression models are shown in Table 5. According to Higgins et al., the intraclass correlation was relatively low (I^2^ = 40.2%) [66], and the moderator LAB genus, pathogen, susceptibility method, pathogen concentration, incubation time, and inoculation volume accounted for 81.3% of the variability between studies (R^2^).

The results demonstrated that *L. monocytogenes* (72.59 ± 1.763 mm, *p* < 0.0001) exhibited significantly higher susceptibility to the LAB genus, as indicated by the higher intercept. This suggests a greater antimicrobial effect of LAB against this pathogen. On the other hand, the ID was lower by −0.889 ± 0.131 mm for *S. aureus*, and by −3.433 ± 0.170 mm for *Salmonella* spp. It is notable that *Enterococcus* (*p* < 0.0001) strains were associated with significantly higher inhibition capacity. Moreover, *Lactococcus* and *Leuconostoc* were not significantly different from one another, yet both with lower inhibition capacity than *Enterococcus* (−3.589 ± 0.431 mm (*p* < 0.0001), and −4.966 ± 0.396 mm (*p* < 0.0001), respectively).

Concerning the susceptibility test method, the disk diffusion method (22.04 ± 0.437, *p* < 0.001) resulted in the highest ID values, while the well diffusion method (16.00 ± 0.437, *p* < 0.001) was significantly linked with lower ID measurements. Pathogen concentration significantly influences the antibacterial effectiveness of LAB against selected bacteria (*p* < 0.001), indicating that as the pathogen concentration increases, the effectiveness of LAB in inhibiting pathogen growth decreases. Additionally, the model evidenced that an increase in incubation time (*p* < 0.001) resulted in a notable positive impact on the ID.

Regarding the inoculation volume nested within the susceptibility test method, similar to the meta-regression findings for *S. aureus*, it was observed that the agar spot method (0.569 ± 0.018, *p* < 0.0001) produced a greater effect on ID values for a given change in inoculated volume, in comparison to the agar disk-diffusion assay (0.344 ± 0.007, *p* < 0.0001). These findings highlight the importance of standardizing inoculum size to obtain reliable, consistent, and comparable results across different methods.

The results also indicated strong evidence of publication bias (*p* = 0.001; Table 5). According to Borenstein et al. and Higgins et al., studies reporting higher effect sizes are more likely to be published than those reporting lower effect sizes, which may introduce publication bias into the analysis [59,126].

**Figure 10:**
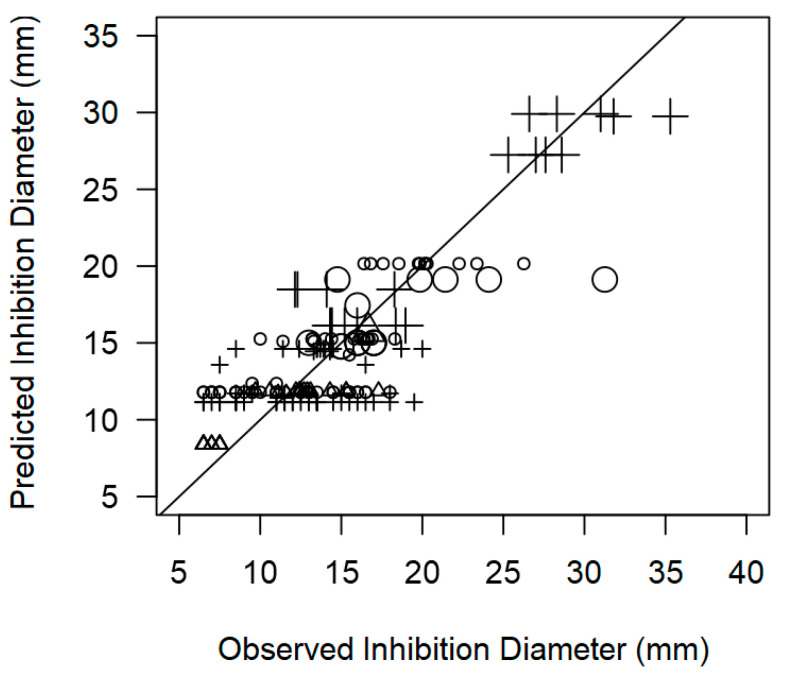
Scatter plot of the observed inhibition diameters produced by lactic acid bacteria against *L. monocytogenes* (○), *Salmonella* spp. (∆) and *S. aureus* (+) versus values predicted by the overarching meta-regression model (R^2^ = 0.827), with 45° reference line. Marker size is proportional to study size.

To evaluate the quality of the meta-regression model, the goodness-of-fit was evaluated by plotting the ID estimates against the observed values (Figure 10). The results revealed a good correspondence against the 45º line. The coefficient of determination (R^2^ = 0.827) was also satisfactory considering the many individual studies from which data were extracted. This indicates that the meta-regression model is robust and could accurately point out the relevant (significant) study characteristics driving the ID outcomes.

## 4. Conclusions

To conclude, this meta-regression analysis highlights the antimicrobial efficacy of LAB against three foodborne pathogens, with *L. monocytogenes* being the most susceptible bacterium to the antimicrobial effects of LAB. The study emphasizes the significant influence of different testing methods—well-diffusion, disk-diffusion, and spot tests—as well as critical factors such as inoculum volume, incubation time, pathogen concentration, and suspension pH in influencing inhibition outcomes. The meta-regression model demonstrated a positive correlation between inoculation volume and incubation time with the inhibition diameter, as larger inoculum sizes and longer incubation times were associated with increased efficacy of LAB. Conversely, pathogen concentration and pH were inversely correlated with inhibition zones, indicating that higher pH levels and pathogen concentrations may reduce the effectiveness of the LAB. These findings underscore the potential of LAB and their metabolites as natural preservatives, providing a promising biopreservation strategy to enhance the microbiological safety of dairy products. Implementing optimized food safety practices and continuous monitoring is essential for ensuring product quality and consumer health.

## Figures and Tables

**Table 1:** Pooled inhibition diameters (mean and standard error in mm) produced by lactic acid bacteria at a pathogen’s inoculum level of 7.0 log CFU/mL and an incubation time of 24 h, as estimated by meta-regression models separately adjusted by foodborne pathogen. Number of observations (*n*), number of primary studies (N) are shown.

Pathogen ^1^	Lactic Acid Bacteria Genus	Assay	Pooled Inhibition Diameter ^2,3^ (SE) [mm]		*n*	N
*L. monocytogenes* ^A^	*Enterococcus*	Overall	20.13 ^A^ (2.21)		40	
		Spot		13.07 ^d^ (1.41)	12	
		Well		20.12 ^b^ (1.24)	28	
	*Lacticaseibacillus*	Overall	21.49 ^A^ (2.65)		34	
		Spot		14.05 ^d^ (1.42)	11	
		Well		24.07 ^b^ (1.32)	23	
	*Lactobacillus*	Overall	20.36 ^A^ (2.65)		45	
		Spot		21.98 ^b^ (1.31)	23	15
		Well		30.73 ^a^ (2.53)	10	
		Disk		13.39 ^d^ (1.40)	12	
	*Lactococcus*	Overall	16.17 ^B^ (2.66)		43	
		Well		16.12 ^c^ (1.32)	42	
	*Leuconostoc*	Overall	17.51 ^B^ (2.68)	-	3	
*S. aureus* ^B^	*Enterococcus*	Overall	19.85 ^A^ (2.07)		35	
		Well		16.20 ^c^ (2.17)	29	
		Disk		16.15 ^c^ (2.79)	6	
	*Lacticaseibacillus*	Overall	21.06 ^A^ (2.06)		41	
		Spot		17.26 ^c^ (2.79)	11	
		Well		23.40 ^b^ (2.11)	24	
		Disk		27.28 ^a^ (2.73)	6	
	*Lactobacillus*	Overall	20.02 ^A^ (2.05)		56	17
		Spot		15.40 ^c^ (1.79)	22	
		Well		22.96 ^b^ (1.10)	22	
		Disk		26.17 ^a b^ (2.73)	12	
	*Lactococcus*	Overall	15.98 ^B^(2.09)		34	
		Spot		18.89 ^b^ (1.36)	34	
	*Leuconostoc*	Overall	17.97 ^B^ (2.08)		5	
		Spot		19.67 ^b^ (2.12)	4	
*Salmonella* ^C^	*Lacticaseibacillus*	Overall	19.06 ^A^ (2.47)		22	
		Spot		14.91 ^b^ (2.31)	3	
		Well		19.31 ^a^ (1.10)	19	
	*Lactobacillus*	Overall	19.93 ^A^ (2.46)		27	
		Spot		13.29 ^b^ (2.32)	15	8
		Well		22.37 ^a^ (1.07)	8	
		Disk		20.30 ^a^ (2.32)	4	
	*Lactococcus*	Overall	11.98 ^B^ (2.57)		12	
		Spot		14.33 ^b^ (1.11)	12	

^1^ Different superscript uppercase letters indicate significant differences in the pooled inhibition diameter between pathogens according to a single meta-regression including pathogen, lactic acid bacteria genus, susceptibility test method, inoculation volume, pathogen concentration and incubation time as moderators. ^2^ Different superscript uppercase letters indicate significant differences in the pooled inhibition diameter between lactic acid bacteria genus by pathogen, according to separate meta-regression models including lactic acid bacteria genus, pathogen concentration and incubation time as moderators. ^3^ Different superscript lowercase letters indicate significant differences in the pooled inhibition diameter between lactic acid bacteria genus by pathogen, according to separate meta-regression models including lactic acid bacteria genus, pathogen concentration, incubation time and susceptibility test method as moderators.

**Table 2:** Final meta-regression model on inhibition diameter produced by lactic acid bacteria (LAB) against *Listeria monocytogenes*, as a function of LAB genus, susceptibility test assay, inoculation volume (μL) and incubation time (h). Number of observations (n) per LAB genus, heterogeneity analysis and *p*-value of the publication bias test are shown.

Parameter	Estimate	Standard Error	*p*-Value	n	Heterogeneity Analysis ^1^
Intercept	11.26	1.69	<0.001		
LAB genus					s^2^ = 43.7
*Lacticaseibacillus*	−6.19	0.55	<0.001	34	τ^2^ = 90.3
*Lactobacillus*	−3.41	0.31	<0.001	45	I^2^ = 67.4%
*Lactococcus*	−5.12	0.61	<0.001	43	τ^2^_res_ = 0.00
*Leuconostoc*	−5.91	0.70	<0.001	3	R^2^ = 99.9%
*Enterococcus*	-	-	-	40	
Method					
Well	−34.89	2.03	<0.001		Publication bias
Disk	−60.15	5.13	<0.001		*p* = 0.509
Inoculation volume	0.316	0.02	<0.001		
Incubation time	0.237	0.06	<0.001		

^1^ Heterogeneity analysis encompasses within-study variability (s^2^), between-study variability (τ^2^), and intra-class correlation (I^2^) of the null model, and residual between-study variability (τ^2^_res_), and between-study variability explained by significant moderators (R^2^) from the full model.

**Table 3:** Final meta-regression model on inhibition diameter produced by lactic acid bacteria (LAB) against *S. aureus*, as a function of LAB genus, pathogen concentration (log CFU/mL), incubation time (h), and inoculation volume (μL) by susceptibility test method. Number of observations (n) per LAB genus, heterogeneity analysis and *p*-value of the publication bias test are shown.

Parameter	Estimate	Standard Error	*p*-Value	n	Heterogeneity Analysis ^1^
Intercept	91.63	2.52	<0.001		
LAB genus					s^2^ = 60.59
*Lacticaseibacillus*	−70.06	2.04	<0.001	41	τ^2^ = 60.05
*Lactobacillus*	−71.82	1.93	<0.001	56	I^2^ = 49.8%
*Lactococcus*	−72.04	1.58	<0.001	34	τ^2^_res_ = 0.00
*Leuconostoc*	−74.59	2.14	<0.001	5	R^2^ = 99.9%
*Enterococcus*	-	-		35	
Pathogen concentration	−16.13	0.33	<0.001		Publication bias
Incubation time	2.24	0.07	<0.001		*p* = 0.001
Inoculation volume					
Agar spot	10.7	0.36	<0.001		
Well diffusion	0.675	0.02	<0.001		

^1^ Heterogeneity analysis encompasses within-study variability (s^2^), between-study variability (τ^2^), and intra-class correlation (I^2^) of the null model, and residual between-study variability (τ^2^_res_), and between-study variability explained by significant moderators (R^2^) from the full model.

**Table 4:** Final meta-regression model on inhibition diameter produced by lactic acid bacteria (LAB) against *Salmonella* spp., as a function of LAB genus, susceptibility test assay, and agar type. Number of observations (n) per LAB genus, heterogeneity analysis and *p*-value of the publication bias test are shown.

Parameter	Estimate	Standard Error	*p*-Value	n	Heterogeneity Analysis ^1^
Intercept	17.88	2.98	<0.001		s^2^ = 39.2
LAB genus					τ^2^ = 52.3
*Lactobacillus*	1.588	1.40	0.255	27	I^2^ = 57.2%
*Lactococcus*	−7.37	1.68	0.006	12	τ^2^_res_ = 23.3
*Lacticaseibacillus*	-	-	-	22	s^2^ = 39.2
Susceptibility method					
Disk	−14.96	6.98	0.032		R^2^ = 55.4%
Well	0.845	4.48	0.850		
Agar					Publication bias
Milk	10.94	8.75	0.211		*p* < 0.001
MRS	9.94	8.75	0.256		
Nutrient	11.91	5.48	0.029		

^1^ Heterogeneity analysis encompasses within-study variability (s^2^), between-study variability (τ^2^), and intra-class correlation (I^2^) of the null model, and residual between-study variability (τ^2^_res_), and between-study variability explained by significant moderators (R^2^) from the full model.

**Table 5:** Overarching meta-regression model on inhibition diameter produced by lactic acid bacteria (LAB) as a function of pathogen, LAB genus, pathogen concentration (log CFU/mL), incubation time (h), and inoculation volume (μL) by susceptibility test method. Number of observations (n) per pathogen and LAB genus, heterogeneity analysis and *p*-value of the publication bias test are shown.

Parameter	Estimate	Standard Error	*p*-Value	n	Heterogeneity Analysis ^1^
Intercept	72.59	1.76	<0.001		
Pathogen					s^2^ = 89.22
*Salmonella*	−3.43	0.17	<0.001	79	τ^2^ = 74.93
*S. aureus*	−0.889	0.13	<0.001	178	I^2^ = 40.2%
*L. monocytogenes*	-	-		165	τ^2^_res_ = 14.00
LAB genus					R^2^ = 81.3%
*Lacticaseibacillus*	−1.84	0.33	<0.001	105	
*Lactobacillus*	−1.96	0.26	<0.001	139	
*Lactococcus*	−3.59	0.43	<0.001	89	Publication bias
*Leuconostoc*	−4.97	0.40	<0.001	8	*p* = 0.001
*Enterococcus*	-	-	-	77	
Method					
Well diffusion	16.00	0.44	<0.001		
Disk diffusion	22.04	0.56	<0.001		
Pathogen concentration	−15.94	0.28	<0.001		
Incubation time	2.23	0.04	<0.001		
Inoculation volume					
Agar spot	0.569	0.02	<0.001		
Disk diffusion	0.344	0.01	<0.001		

^1^ Heterogeneity analysis encompasses within-study variability (s^2^), between-study variability (τ^2^), and intra-class correlation (I^2^) of the null model, and residual between-study variability (τ^2^_res_), and between-study variability explained by significant moderators (R^2^) from the full model; n: number of observations.

## Data Availability

No new data were created or analyzed in this study. Data sharing is not applicable to this article.
